# ADOPTING A STRENGTH-BASED, PERSON-CENTERED RISK ASSESSMENT CLINICAL DECISION SUPPORT TOOL: WHAT ARE THE BENEFITS?

**DOI:** 10.1093/geroni/igad104.3163

**Published:** 2023-12-21

**Authors:** Heather MacLeod, Véronique Provencher, Dorothy Kessler, Mary Egan, Dominique Giroux, Marie-Jeanne Kergoat, Lewis Krystina, Veillette Nathalie

**Affiliations:** Perley Health, Ottawa, Ontario, Canada; Université de Sherbrooke, Sherbrooke, Quebec, Canada; Queen’s University, Kingston, Ontario, Canada; University of Ottawa, Ottawa, Ontario, Canada; Université Laval, Québec, Quebec, Canada; Université de Montréal, Montréal, Quebec, Canada; University of Ottawa, Ottawa, Ontario, Canada; Université de Montréal, Montréal, Quebec, Canada

## Abstract

Most older adults want to age in place even if changes in their health results in home safety concerns. A consistent approach to assessing both the physical and psychological risks associated with the decision to remain at home is lacking. The Living with Risk: Decision Support Approach (LwR:DSA) is a recently validated innovative clinical tool that supports a balanced, systematic and person-centered assessment of risks, by analyzing their negative and positive consequences. The aim of this mixed-method study was to understand the barriers and facilitators to using the LwR:DSA during usual care to determine how best to support widespread adoption. Twenty-two hospital- and community-based clinicians used the LwR:DSA for eight weeks. Individual interviews were performed to document the factors that hindered and helped the use of the LwR:DSA in their clinical setting. The interviews were analyzed using Qualitative Description and the positive impact of using the LwR:DSA emerged as one of the facilitator themes. The participants described that using the LwR:DSA in practice improved their clinical decision making, communication and their ability to provide person-centered care. More specifically, the LwR:DSA 1) helped the clinicians understand the risk level and the context, causes, and consequences of the safety concern; 2) guided them to co-create with the older adult, agreeable recommendations to reduce the risk of adverse outcomes; 3) decreased clinician discomfort and 4) supported authentic conversations with the older adult. Understanding the benefits of using the LwR:DSA provides key information for clinicians by challenging the status quo of their current practice.

